# Bacteriophages affect evolution of bacterial communities in spatially distributed habitats: a simulation study

**DOI:** 10.1186/s12866-015-0620-4

**Published:** 2016-01-27

**Authors:** Alexandra Igorevna Klimenko, Yury Georgievich Matushkin, Nikolay Alexandrovich Kolchanov, Sergey Alexandrovich Lashin

**Affiliations:** Institute of Cytology and Genetics SB RAS, Lavrentiev Avenue 10, Novosibirsk, 630090 Russia; Novosibirsk State University, Pirogova st. 2, Novosibirsk, 630090 Russia

**Keywords:** Microbial community, Bacteria, Archaea, Bacteriophage, Phage, Ecological simulation, Evolution, Evolutionary modeling, Prokaryotes

## Abstract

**Background:**

Bacteriophages are known to be one of the driving forces of bacterial evolution. Besides promoting horizontal transfer of genes between cells, they may induce directional selection of cells (for instance, according to more or less resistance to phage infection). Switching between lysogenic and lytic pathways results in various types of (co)evolution in host-phage systems. Spatial (more generally, ecological) organization of the living environment is another factor affecting evolution. In this study, we have simulated and analyzed a series of computer models of microbial communities evolving in spatially distributed environments under the pressure of phage infection.

**Results:**

We modeled evolving microbial communities living in spatially distributed flowing environments. Non-specific nutrient supplied in the only spatial direction, resulting in its non-uniform distribution in environment. We varied the time and the location of initial phage infestation of cells as well as switched chemotaxis on and off. Simulations were performed with the Haploid evolutionary constructor software (http://evol-constructor.bionet.nsc.ru/).

**Conclusion:**

Simulations have shown that the spatial location of initial phage invasion may lead to different evolutionary scenarios. Phage infection decreases the speciation rate by more than one order as far as intensified selection blocks the origin of novel viable populations/species, which could carve out potential ecological niches. The dependence of speciation rate on the invasion node location varied on the time of invasion. Speciation rate was found to be lower when the phage invaded fully formed community of sedentary cells (at middle and late times) at the species-rich regions. This is especially noticeable in the case of late-time invasion.

Our simulation study has shown that phage infection affects evolution of microbial community slowing down speciation and stabilizing the system as a whole. This influencing varied in its efficiency depending on spatially-ecological factors as well as community state at the moment of phage invasion.

**Electronic supplementary material:**

The online version of this article (doi:10.1186/s12866-015-0620-4) contains supplementary material, which is available to authorized users.

## Background

Bacteriophages are known to be one of the driving forces of bacterial evolution [1–3]. It is generally thought that phages are responsible for about 10–50 % of the total bacterial mortality in surface waters, and 50–100 % in environments that are unfriendly to protists, such as low-oxygen lake waters [4]. Besides promoting horizontal transfer of genes between cells [5–7], they may induce either directional selection of cells [8], for instance, according to more or less resistance to phage infection [9–12], or fluctuating selection [10, 13]. Switching between lysogenic and lytic pathways results in various types of (co)evolution in bacterial cell-phage systems [14, 15]. Spatial (more generally, ecological) organization of the living environment is another factor affecting evolution [16–18]. A number of studies, both experimental and theoretical have recently been published on various aspects of phage-bacteria evolution.

The population mixing increasing host exposure to phages via selection for greater resistance and infectivity ranges was proved to promote arms-race dynamics [13]. It explains the variation in coevolutionary dynamics between different host–parasite systems, and more specifically the observed discrepancies between laboratory and field bacteria–virus coevolutionary studies [13]. Using a spatially explicit, individual-based model, it was shown that less infective pathogens may have an advantage in spatially structured populations, even when well-mixed models predict that they will not [19].

In a time-shift experiment with both sympatric and allopatric phages from either contemporary or earlier points in the season, it was demonstrated that bacterial resistance is higher against phages from the past, regardless of spatial sympatry or how much earlier in the season phages were collected [20]. It was also shown that future bacterial hosts are more resistant to both sympatric and allopatric phages than contemporary bacterial hosts. Nutrients availability was both theoretically and experimentally shown to affect the relative extent of escalation of resistance and infectivity (arms race dynamic) and fluctuating selection (fluctuating selection dynamic) in experimentally coevolving populations of bacteria and viruses [21]. Scanlan and colleagues have shown that in addition to affecting genome-wide evolution in loci not obviously linked to parasite resistance, coevolution can also constrain the acquisition of mutations beneficial for growth in the abiotic environment [22]. The conditions for achieving coexistence on the edge between two habitats, one of which is a bacterial refuge with conditions hostile to phage whereas the other is phage friendly were theoretically studied in [23]. They analyzed how bacterial density-dependent, or quorum-sensing, mechanisms such as the formation of biofilm can produce such refuges and edges in a self-organized manner.

A multiscale model of dynamic coevolution between hosts and viruses in an ecological context incorporating CRISPR immunity principles was presented in [24]. Hosts and viruses were shown to coevolve to form highly diverse communities. They observed evolutionary dynamics consistent with both incomplete selective sweeps of novel strains (as single strains and coalitions) and the recurrence of previously rare strains. Coalitions of multiple dominant host strains were predicted to arise because host strains can express nearly identical immune phenotypes mediated by CRISPR defense albeit with different genotypes [24]. The evolution of generalism in well-mixed populations was found to be highly sensitive to the severity of associated fitness costs, but the constraining effect of costs on the evolution of generalism is lessened in spatially structured populations [25]. The contrasting outcomes between the two environments was explained by different scales of competition (i.e., global vs. local). They suggested that local interactions may have important effects on the evolution of generalism in host-parasite interactions, particularly in the presence of high fitness costs [25].

In our previous study [26] we modeled the opposite trends of genome amplification/simplification occurred in microbial communities via gene horizontal transfer/gene loss. It was demonstrated that species with reduced genomes tend to replace genetically and metabolically rich species under highly favorable environmental conditions. Under unfavorable conditions, the opposite tendency was observed. It was also shown that phages invasion into the system radically changed the current evolutionary trends.

A wide range of mathematical and computational techniques is used for modeling and simulation of phage-bacterium systems. There are both classical ODE/PDE approaches (see examples in [11, 27–31]) and modern agent-based and/or multiscale approaches (see examples in [19, 23, 25, 32, 33]).

In this study, we have built and simulated a series of computer models of microbial communities evolving in spatially distributed environments under the pressure of phage infection. Communities inhabited spatially distributed flowing environments. Non-specific nutrient supplied in the only spatial direction, resulting in its non-uniform distribution in environment. We varied the time and the location of initial phage invasion as well as switched chemotaxis on and off and observed that these factors may lead to different evolutionary scenarios.

## Methods

### Model overview

**Fig. 1:**
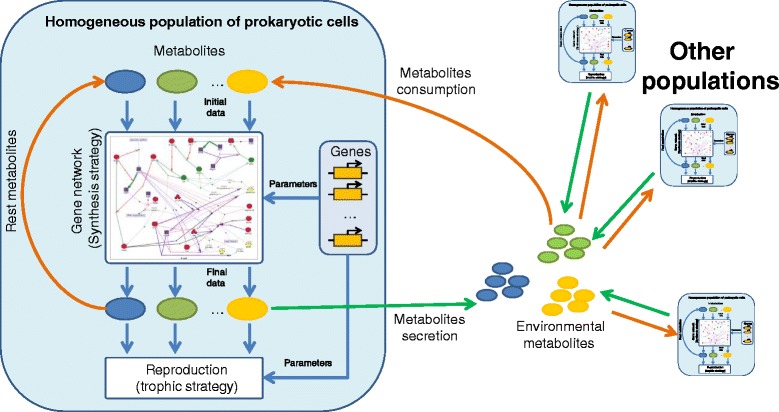
The hierarchical scheme of the HEC model (according to [35, 38])

**Fig. 2:**
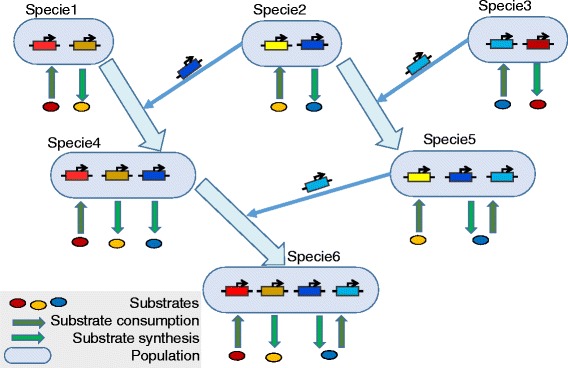
Simulation of horizontal gene transfer in the HEC (according to [40])

We have used the Haploid evolutionary constructor (HEC) [34, 35] to build the model and to perform the simulations. The HEC models microbial community consisting of different microbial species which we call populations. The hierarchical scheme of HEC models is shown in Fig. 1. Each population consists of cells sharing the same metabolic specificity i.e. nutrients consumed and products secreted. We assume that metabolic pathways of substrates utilization and products synthesis controlled by corresponding gene networks are the same for all cells of a population. Therefore, we consider genes just as numerical parameters of these gene network models. The parameters may be responsible for efficiency of either utilization or synthesis of metabolites. Note that the HEC architecture provides various mathematical formalisms to be used to describe a gene network model mathematically (see details in [35]). In this case, mutations just change the numerical value of a certain parameter in one or several cells (which is interpreted as an origin of novel allelic variant) resulting in genetic polymorphism in a population. On the other hand, selection of more fit allelic combinations leads to the extinction of weaker alleles decreasing the polymorphism. Horizontal transfer of genes between cells of different populations is modeled in HEC via rearrangement of recipient cell’s gene network by embedding additional nodes into this network (Fig. 2). Loss of genes is modeled in the same manner, nodes, of course, are deleted. We suppose in our model, that such events of horizontal gene transfer and gene loss are associated with the origin of novel species as they significantly change the metabolic properties of cells. Technically, we use in HEC the super-individual concept [36, 37].

### Spatially distributed habitats

**Fig. 3:**
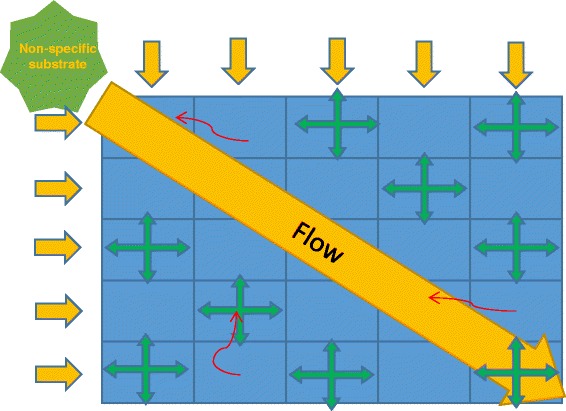
Spatial organization of the habitat. Mesh of 5х5 nodes. The flow determines the gradient of non-specific substrates. Green arrows depict the directions of diffusion. Red arrows show an example of chemotactic behavior of cells

We describe the spatial organization of environment by a grid of so-called point environments or nodes – small well-mixed volumes containing cells, phage particles and substances. These volumes exchange their contents affected by diffusion and flow as well as during the bacterial chemotaxis activity (Fig. 3, detailed description in [38]). Flow is a directed force mixing contents of the spatial system and drawing particles from its beginning to the end. On the contrary diffusion is considered as undirected dissemination of the point environment’s contents. Chemotaxis is an ability of bacterial cells to move towards the attractants and away from repellents, i.e. a capacity to move to a habitat with more chemically beneficial conditions. Such a grid of multiple well-mixed volumes is capable to simulate heterogeneous distributions of cells, substances and phage particles.

We considered 2D spatially distributed flowing habitat represented as a 5x5 grid (Fig. 3). In-flow supplies the habitat with non-specific nutrients we call non-specific substrates through the upper and leftmost nodes. The flow and diffusion then spread nutrients over all other nodes of the habitat including not only non-specific substrates, but also metabolites synthesized and secreted by cells (specific substrates). Cells themselves are also passively transported by the flow and diffusion, but additionally may move via chemotaxis (details in [38]). Thus, the (1,1) node and its neighbors may be considered as nutrient-rich (in terms of non-specific substrates), while the (5,5) node and its neighbors are nutrient-poor (in the same terms). Here and after let us call the (1,1) node as the “top”, the (3,3) – “middle”, and the (5,5) – “bottom” of the habitat. It should be noted that we have tested the 10 × 10 and 20 × 20 habitats and found no principal difference with the 5 × 5 case.

### Modeling bacteriophage infection

In the HEC, bacteriophages (phages) are described using special type of populations – phage populations, which are capable to infect microbial cells. In this particular case the phage was able to infect those cells which are capable to utilize S1 substrate. The infection process includes the following stages:

1. infestation of healthy cells by phage invasion (from environment into some cells of a population);
2. phages reproduction inside the infected cells;
3. phage burst after cell lysis.

Infected cells may develop then according to either lytic or lysogenic pathway. In the first case, the infected cells die bursting new phages into the habitat. In the second case, on the contrary, no cell die, phage genes are integrated into cell genome and the cells become prophages. The choice of lysogenic or lytic scenarios depends on environmental conditions and cells well-being at the moment of infestation (it is in accordance with biologically known facts [39]). Under favorable conditions, in the case of a positive population dynamics ensured by high environmental substrate concentrations and/or better genetic adaptation compared to other populations, the infection process occurs along the lytic pathway. Otherwise, under unfavorable conditions, infection takes the lysogenic pathway. Later, if the conditions are improved, a part of the population may switch to the lytic pathway, which is followed by the death of this part of the population and generation of phages. The detailed description of the modeling technique for phages in the HEC is presented in [26]. In this study, we considered invasion of phages varying both the moment of invasion and its localization.

## Results and discussion

**Fig. 4:**
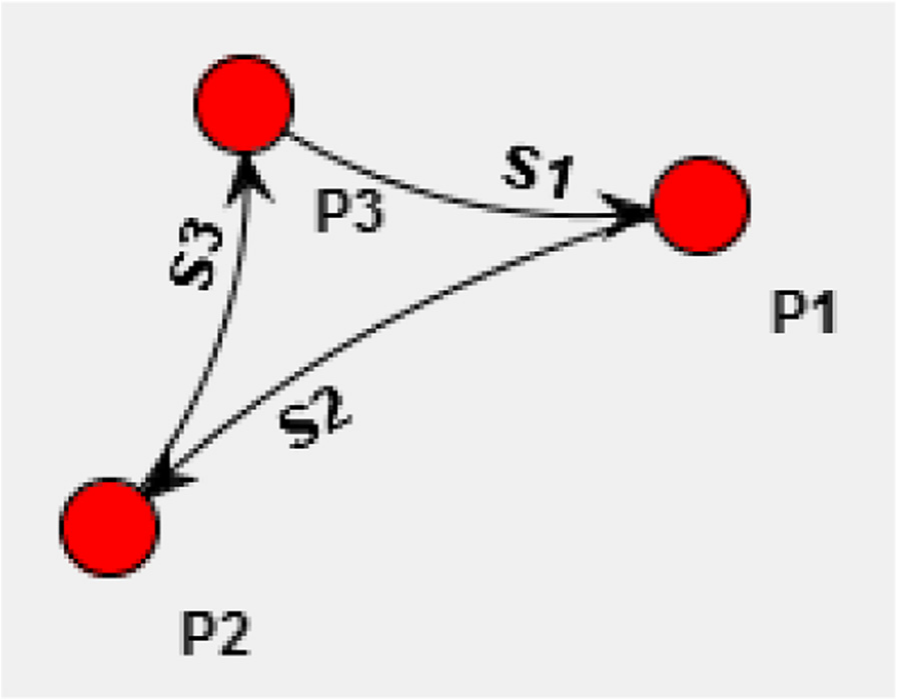
Trophic graph of the initial community. N1 – non-specific substrate consumed by all populations (P1, P2, P3) of the community. S1, S2, S3 – specific substrates synthesized by corresponding cells. $P1\overset{S2}{\to }P2$ means that cells of P1 population produce S2 substrate, which is consumed by cells of P2 population

Using the approach described above, we have developed a model of spatially distributed microbial community under bacteriophage attack. We started simulations with the simple symbiotic community of three different populations (Fig. 4) uniformly inhabited spatially distributed 2D environment (Fig. 3).

In the course of evolution the processes of horizontal gene transfer and gene loss stochastically simulated. It was associated with the origin of novel species (see details in the previous chapter). Initial environmental conditions promoted the origin of species possessing more and more complex genomes built combinatorically from relatively simple genomes of initial cells, as it was previously studied in [40]. We varied the time of initial phage invasion as well as its localization. The main questions we wanted to discover were the following:

- how does the phage invasion affect the ecological-evolutionary trends taking course in the community?
- what is the role played by spatial factors in found effects?
- is the bacteriophage an impediment for microbial communities’ evolution?

**Fig. 5:**
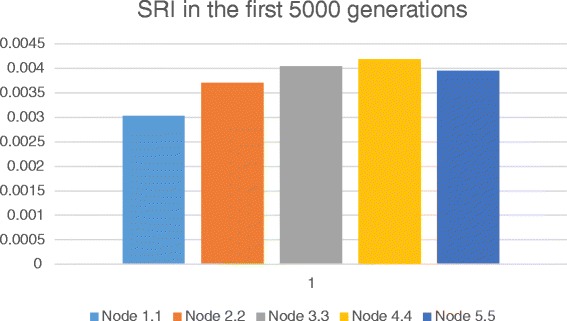
SRI calculated for the first 5000 generations in various nodes

Varying the time of initial phage invasion, we have found that infestation in general inhibits speciation, and after a certain time the species composition and the size of the community become stationary (Fig. 5, Additional file: 1). It looks as if the phages stop the evolution of the community. It should be noted that we talk about speciation in terms of variation of gene set involved in metabolic networks and the evolution of immune mechanisms is out of scope in this simulation study. It explains why we observe decrease in a speciation rate under phage invasion conditions whereas other studies [2] putting an emphasis on the evolution of defense and counter-defense strategies provide evidences of increasing phage and bacterial evolution rate. The other possible reason of the discrepancy is because we focused primarily on interactions between bacteria and temperate phages, rather than lytic phages as it has been done in the above-mentioned case.

We obtained the same results when chemotaxis was switched on/off. This effect has been estimated using the Speciation Rate Index (SRI), which was calculated according to the following formula (see also script file in Additional files):

1 $$ SRI=\frac{\parallel \left\{\left.p\right|p\mathit{\hbox{-}} newly\kern0.5em  emerged\kern0.5em  viable\kern0.5em  populations\right\}\parallel }{time\kern0.5em  span} $$

where time_span is the period speciation rate is estimated about; a population is assumed to be viable if it survived over a period of thres generations (where thres is a threshold value, in our case, 500). According to the equation 1, the higher SRI is, the more intensive the speciation is over this time period. Conversely, the lower SRI is, the fewer viable species originate in time.

### Chemotaxis-off case

**Fig. 6:**
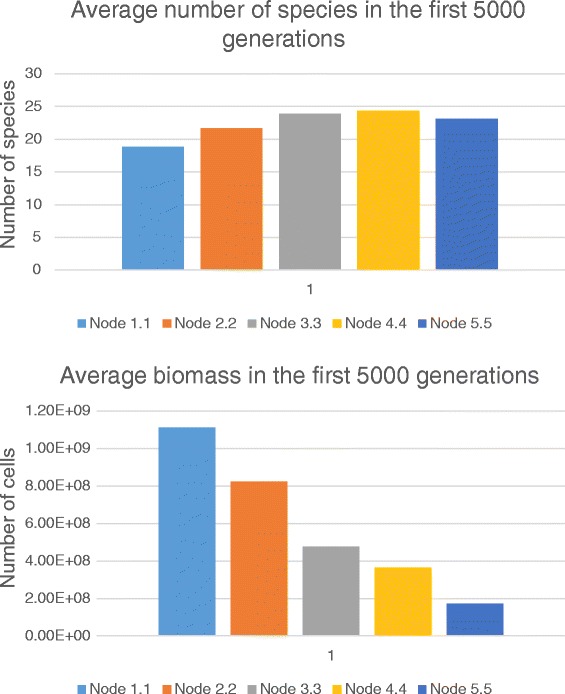
Average number of species (up) and biomass (down) over the first 5000 generations in various nodes (see also the script in Additional file 3)

Initially, we have estimated SRI in all spatial nodes of the habitat in the absence of phage invasion (Fig. 5). Fig. 5 shows that speciation rate growth according to moving away from the in-flow (top) nodes. It could be explained by the fact that in top nodes there are several populations, which effectively utilize the non-specific substrate. These population have got dominate biomass. At the same time, novel ecological licenses associated with high concentrations of specific substrates arise in middle and top nodes due to transportation via the flow. It opens possibilities for fixation of novel species specialized on utilization of those specific substrates. That is to say, in distant, bottom nodes, higher biodiversity is associated with low biomass (Fig. 6).

After that, we have analyzed how the phage invasion affects speciation rate and species richness. Results obtained show the dependency of speciation rate on invasion parameters (Table 1).

**Table 1:** Average SRI values before and after phage invasion in various habitat locations (see Additional file 4). First 1000 generations has not been taken into account as there is a bias associated with high initial speciation

	Early-time (1st generation)		Middle-time (5000th generation)		Late-time (6600th generation)	
Into node (1,1)	SRI before	SRI after	SRI before	SRI after	SRI before	SRI after
	-	0.856e-3	6.850e-3	0.520e-3	7.143e-3	0.529e-3
Into node (3,3)	SRI before	SRI after	SRI before	SRI after	SRI before	SRI after
	-	0.744e-3	7.275e-3	0.400e-3	7.946e-3	0.206e-3
Into node (5,5)	SRI before	SRI after	SRI before	SRI after	SRI before	SRI after
	-	0.578e-3	8.250e-3	0.600e-3	6.964e-3	0.412e-3

Table 1 shows that phage invasion results in decrease of speciation rate by more than one order. As is easy to see the time dependency of speciation rate varies with the distance between the node and in-flow location:

1. In the case of early-time invasion, there is an inverse dependence – the farther the place of invasion, the less the speciation rate after infestation (Fig. 7).

**Fig. 7:**
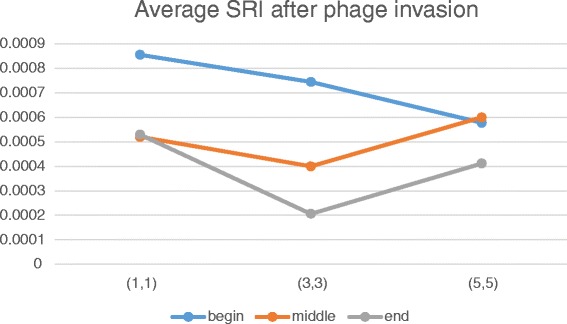
Dependence of SRI on time of invasion and invasion initial localization. Blue plot – early-time (1st generation), orange – middle-time (5000th generation), grey – late-time (6600th generation). X-axis – number of the node of initial invasion, Y-axis – average SRI calculated up to the end of simulation
2. In the case of middle-time invasion, the lowest speciation rate is observed when the phage invades the central node (3,3) of the habitat. It is notable that if the phage invades the most bottom node (5,5), then average SRI becomes higher compared to the most top node (1,1).
3. In the case of late-time invasion, results look similar to the previous case except that average SRI is lower when the phage invaded central and bottom nodes.

As it was previously said, distant nodes are characterized by higher concentrations of the specific substrates. Therefore, these results are in accordance with earlier reported [41] suggestion that the viral effect is probably larger in eutrophic waters than in oligotrophic waters.

Table 2 shows that in the case of middle-time invasion, average SRI increases from top (1,1) nodes (non-specific substrate-rich) to bottom (5,5) nodes (non-specific substrate-poor). Additional plots (see Additional file : 2) show that the phage invasion leads to drastic reduction of species richness of the community. The data confirm that SRI as well as species richness are higher in the absence of phages and when there are many ecological licenses.

**Table 2:** Average SRI calculated over the whole simulation time with respect to the initial invasion location (see Additional file 4)

	Early-time (1st generation)	Middle-time (5000th generation)	Late-time (6600th generation)
Into node (1,1)	1.3030e-3	5.6061e-3	6.5657e-3
Into node (3,3)	1.1717e-3	5.8687e-3	7.2121e-3
Into node (5,5)	1.2828e-3	6.3333e-3	6.8081e-3

### Chemotaxis-on case

**Fig. 8:**
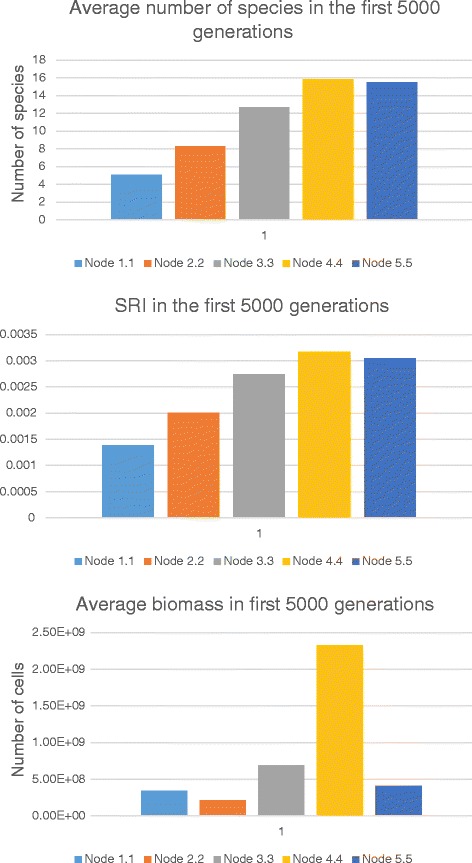
Distributions of average values for (a) species richness; (b) SRI; (c) total biomass in spatial nodes. Values were calculated for the first 5000 generations

In cases when chemotaxis is switched on (see Additional file 5), the spatial stratification of a community in terms of biomass is more expressed. We hypothesize that this is due to the observed accumulation of specific substrates in nodes close to the bottom of the habitat. Anyway, the distributions of species richness and SRI in general look similarly to the corresponding distributions obtained for the previous case. However, the spatial differences here are more evident (Fig. 8). On the other hand, the total species number as well as SRI in this case (chemotaxis is on) are definitely lower compared to the previous case (chemotaxis is off). It is in good agreement with evolutionary biology data postulating that migration impedes speciation while isolation promotes it [42–44].

**Fig. 9:**
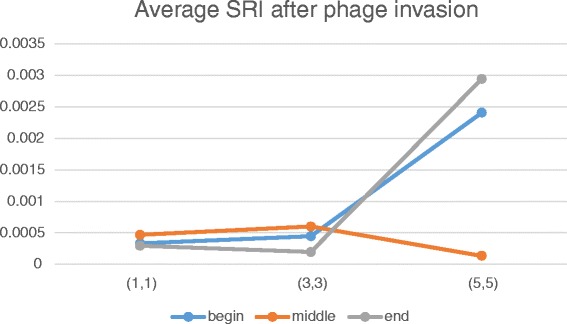
Dependence of SRI on time of invasion and invasion initial localization (chemotaxis is on). Blue plot – early-time (1st generation), orange – middle-time (5000th generation) , grey – late-time (6600th generation). X-axis – number of the node of initial invasion, Y-axis – average SRI calculated up to the end of simulation (built according to data from Additional file 5)

Figure 9 shows that phage invasion into the bottom node (5,5) led to the growth of speciation rate both for early-time and late-time cases. It is related to the observed fact that the phage infection could not fixate in the habitat when invaded into bottom nodes. However, in the chemotaxis-on case, if the phage invaded top nodes, the species richness observed to be higher in bottom nodes.

## Conclusions

In this simulation study, we have shown that bacteriophages may act as constraining factors of microbial community evolution. Phage infection decreases the speciation rate by more than one order as far as intensified selection blocks the origin of novel viable populations/species, which could carve out potential ecological niches. At the same time, phages act as a stabilizing factor suspending superfluous speciation and encouraging stationary state of the system (in terms of species number).

It has also been shown that the dependence of speciation rate on the invasion node location varied on the time of invasion. Speciation rate is found to be lower when the phage invaded fully formed community (middle and late times) at the species-rich nodes (central node (3,3)). This is especially noticeable in the case of late-time invasion.

Those dependencies differ in the case of chemotaxis switched on. In both early-time and late-time cases, the speciation rate remains relatively high when phage invaded bottom nodes. This phenomenon requires additional investigations.

As a result, our simulation study shows that phage infection affects evolution of microbial community slowing down speciation and stabilizing the system as a whole. This influence varied in its efficiency depending on spatially-ecological factors as well as community state at the moment of phage invasion.

## Additional files

Additional file 1: 
**Data processing script addZeros.sce**. A Scilab script for data preprocessing of popSize.txt files that is necessary for some other scripts (such as speciation_rate.sce). (SCE 2.71 kb) Additional file 2: 
**Data processing script speciation_rate.sce**. A Scilab script that calculates Speciation Rate Index (SRI). (SCE 13.7 kb) Additional file 3:
**Data processing script averageSRandBiomass.sce.** A Scilab script that calculates averaged by time species richness (number of species) and total biomass of the community. (SCE 9.20 kb) Additional file 4:
**SRI.SR.Biomass.chemoff.xlsx.** An Excel spreadsheet containing the data on species richness, SRI and biomass for the chemotaxis-off cases. (XLSX 38.9 kb) Additional file 5:
**SRI.SR.Biomass.chemon.xlsx.** An Excel spreadsheet containing the data on species richness, SRI and biomass for the chemotaxis-on cases. (XLSX 36.5 kb) Additional file 6:
**Phage.extinction.chemon.xlsx.** An Excel spreadsheet containing the data on the extinction of phage populations for the chemotaxis-on cases. (XLSX 8.98 kb)

## Abbreviations

HEC: Haploid evolutionary constructor software
SRI: Speciation rate index
