# Erratum to: Bacteriophages affect evolution of bacterial communities in spatially distributed habitats: a simulation study

**DOI:** 10.1186/s12866-016-0677-8

**Published:** 2016-04-14

**Authors:** Alexandra Igorevna Klimenko, Yury Georgievich Matushkin, Nikolay Alexandrovich Kolchanov, Sergey Alexandrovich Lashin

**Affiliations:** Institute of Cytology and Genetics SB RAS, Lavrentiev Avenue 10, Novosibirsk, 630090 Russia; Novosibirsk State University, Pirogova st. 2, Novosibirsk, 630090 Russia

Unfortunately, the original version of this article [1] had the below errors related to the additional files, extra supplementary material and their citations.

- Additional files 1, 2, 3, 4, 5 and 6 listed in the article only open to an empty word document. Please see these as additional files to this erratum.
- Figure 4 was incorrect. Please see the correct figure below.

**Fig. 4:**
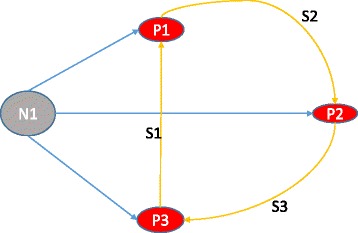
Trophic graph of the initial community. N1 – non-specific substrate consumed by all populations (P1, P2, P3) of the community. S1, S2, S3 – specific substrates synthesized by corresponding cells. *P*1 → _*S*2_ *P*2 means that cells of P1 population produce S2 substrate, which is consumed by cells of P2 population
- "(Fig. 5, Additional file: 1)" in the text should have been “(Fig. 5, Additional file: 7 (Table S1, S3))”
- In the text “ Additional plots (see Additional file: 2) show that the phage invasion leads to drastic reduction of species richness of the community.” should be “Additional plots (see Additional file: 7 (Table S2)) show that the phage invasion leads to drastic reduction of species richness of the community. The same is true for chemotaxis-on case (see Additional file 7: (Table S4)).”
- There should have been an Additional file 7 with the four supplementary tables included. Please see this additional file as an additional file to this erratum.
- There should have been an Additional file 8 which contained 12 extra supplementary files and a word document of the related captions to these. In the text this should have been cited within the text. The text “It should be noted that we have tested the 10 × 10 and 20 × 20 habitats and found no principal difference with the 5 × 5 case.” should have been "It should be noted that we have tested the 10 × 10 and 20 × 20 habitats and found no principal difference with the 5 × 5 case. For the HEC scripts used for building models and other used Scilab scripts to be acquainted with see Additional file 8". Please see this additional file as an additional file to this erratum.

## Additional files

Additional file 1: Data processing script addZeros.sce. A Scilab script for data preprocessing of popSize.txt files that is necessary for some other scripts (such as speciation_rate.sce). (SCE 2 kb) Additional file 2: Data processing script speciation_rate.sce. A Scilab script that calculates Speciation Rate Index (SRI). (SCE 13 kb) Additional file 3: Data processing script averageSRandBiomass.sce. A Scilab script that calculates averaged by time species richness (number of species) and total biomass of the community. (SCE 9 kb) Additional file 4: SRI.SR.Biomass.chemoff.xlsx. An Excel spreadsheet containing the data on species richness, SRI and biomass for the chemotaxis-off cases. (XLSX 38 kb) Additional file 5: SRI.SR.Biomass.chemon.xlsx. An Excel spreadsheet containing the data on species richness, SRI and biomass for the chemotaxis-on cases. (XLSX 36 kb) Additional file 6: Phage.extinction.chemon.xlsx. An Excel spreadsheet containing the data on the extinction of phage populations for the chemotaxis-on cases. (XLSX 8 kb) Additional file 7: Table S1. Population dynamics in the total volume for the models varying by the time and the location of initial phage infestation of cells Chemotaxis is off. **Table S2.** Species richness (number of species) in the total volume for the models varying by the time and the location of initial phage infestation of cells Chemotaxis is off. **Table S3.** Population dynamics in the total volume for the models varying by the time and the location of initial phage infestation of cells Chemotaxis is on. **Table S4.** Species richness (number of species) in the total volume for the models varying by the time and the location of initial phage infestation of cells Chemotaxis is on. (DOCX 1206 kb) Additional file 8: S1 Archive containing the HEC executable file. 7-Zip archive containing the HEC executable file (Windows version). To switch chemotaxiss on pass 0.1 as a second command line parameter (the first is a model script). **S2.** HEC script 2D_phage.plentiful_edge.early.txt of early-time phage into (1,1) model. Text file with the model script of early-time phage added into (1,1) model. **S3.** HEC script 2D_phage.center.early.txt of early-time phage into (3,3) model. Text file with the model script of early-time phage added into (3,3) model. **S4.** HEC script 2D_phage.poor_edge.early.txt of early-time phage into (5,5) model. Text file with the model script of early-time phage added into (5,5) model. **S5.** HEC script 2D_phage.plentiful_edge.middle.txt of middle-time phage into (1,1) model. Text file with the model script of middle-time phage added into (1,1) model. **S6.** HEC script 2D_phage.center.middle.txt of middle-time phage into (3,3) model. Text file with the model script of middle-time phage added into (3,3) model. **S7.** HEC script 2D_phage.poor_edge.middle.txt of middle-time phage into (5,5) model. Text file with the model script of middle-time phage added into (5,5) model. **S8.** HEC script 2D_phage.plentiful_edge.late.txt of late-time phage into (1,1) model. Text file with the model script of late-time phage added into (1,1) model. **S9.** HEC script 2D_phage.center.late.txt of late-time phage into (3,3) model. Text file with the model script of late-time phage added into (3,3) model. **S10.** HEC script 2D_phage.poor_edge.late.txt of late-time phage into (5,5) model. Text file with the model script of late-time phage added into (5,5) model. **S11.** Data processing script speciesRichnessFluctuation.sce. A Scilab script that plots species richness dynamics. **S12.** A library SRlib.sce. A Scilab library sources used by such scripts as speciesRichnessFluctuation.sce, averageSRandBiomass.sce and speciation_rate.sce. (ZIP 594 kb)
