# Supplementary material for: Erratum to: Bacteriophages affect evolution of bacterial communities in spatially distributed habitats: a simulation study
Source: BMC Microbiol. 2016 Apr 14;16:67. doi: 10.1186/s12866-016-0677-8 (PMC4831195; doi:10.1186/s12866-016-0677-8)
Supplement: Additional file 7: Table S1. — Population dynamics in the total volume for the models varying by the time and the location of initial phage infestation of cells Chemotaxis is off. Table S2. Species richness (number of species) in the total volume for the models varying by the time and the location of initial phage infestation of cells Chemotaxis is off. Table S3. Population dynamics in the total volume for the models varying by the time and the location of initial phage infestation of cells Chemotaxis is on. Table S4. Species richness (number of species) in the total volume for the models varying by the time and the location of initial phage infestation of cells Chemotaxis is on. (DOCX 1206 kb) [file 12866_2016_677_MOESM7_ESM.docx]

Tables

Table S1

Population dynamics in the total volume for the models varying by the time and the location of initial phage infestation of cells. Chemotaxis is off (see Additional files S2-S10).

|  | Early-time  (1^st^ generation) | Middle-time  (5000^th^ generation) | Late-time  (6600^th^ generation) |
| --- | --- | --- | --- |
| Into node (1,1) |  |  |  |
| Into node (3,3) |  |  |  |
| Into node (5,5) |  |  |  |

Table S2

Species richness (number of species) in the total volume for the models varying by the time and the location of initial phage infestation of cells. Chemotaxis is off (see Additional files S2-S10. Plots are made using the script from Additional files S11-S12).

|  | Early-time  (1^st^ generation) | Middle-time  (5000^th^ generation) | Late-time  (6600^th^ generation) |
| --- | --- | --- | --- |
| Into node (1,1) | 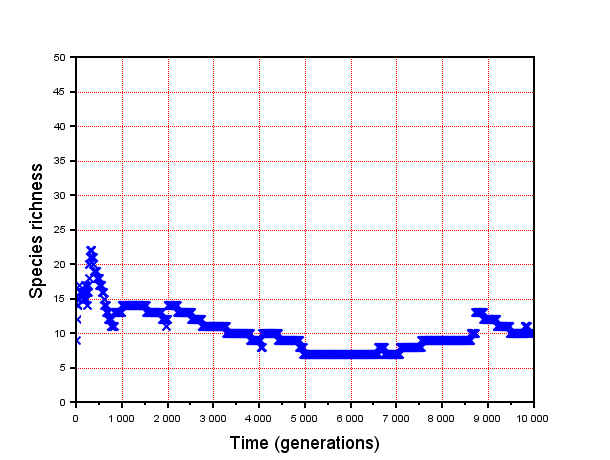 | 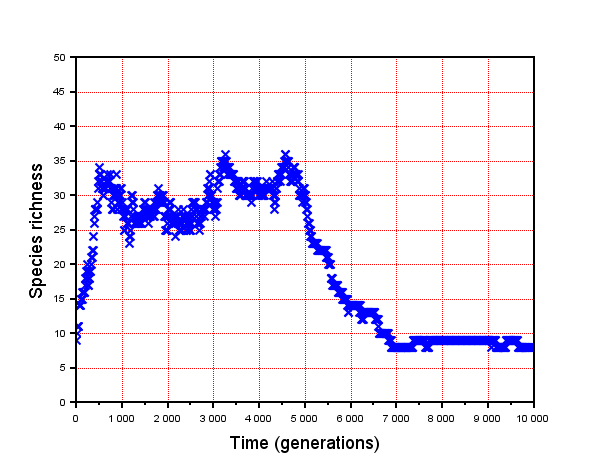 | 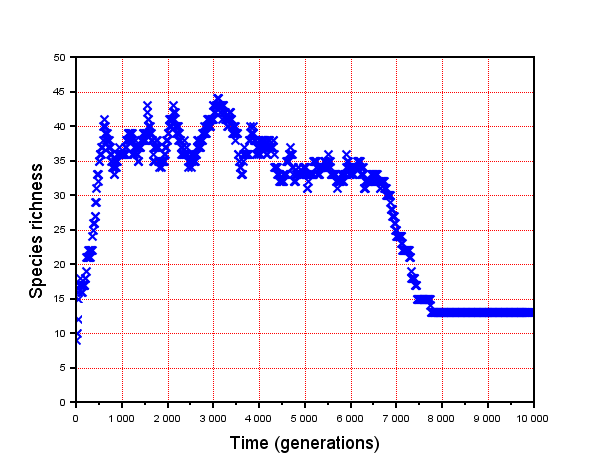 |
| Into node (3,3) | 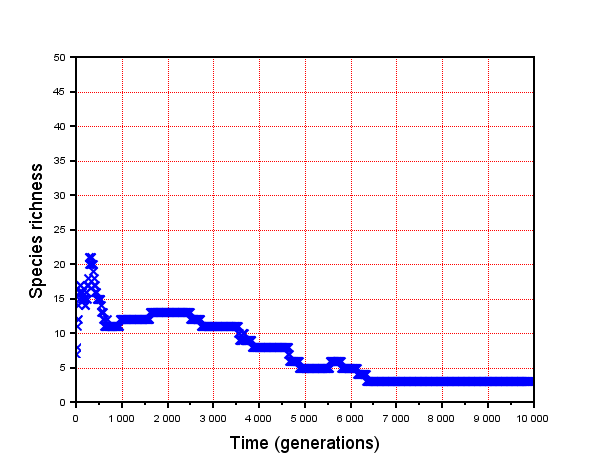 | 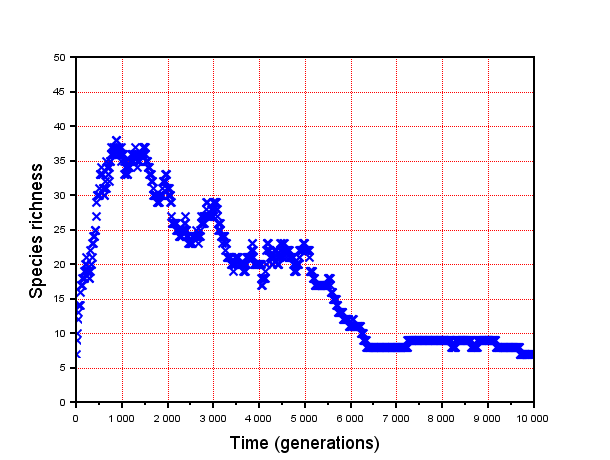 | 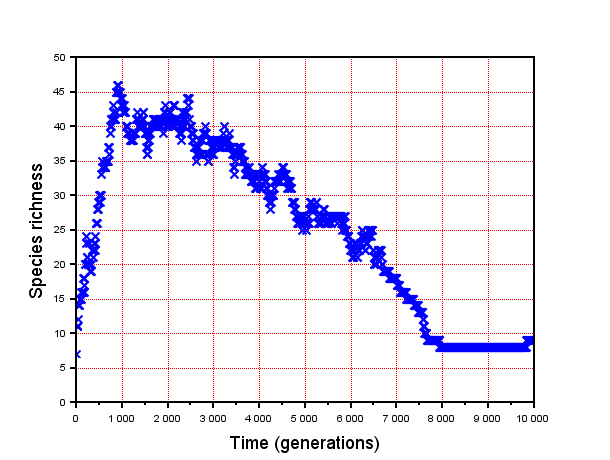 |
| Into node (5,5) | 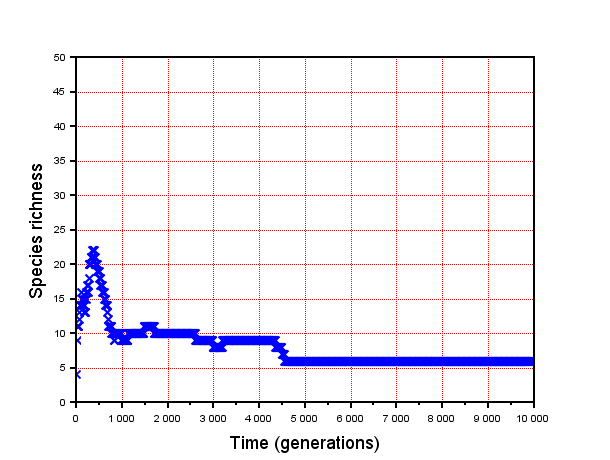 | 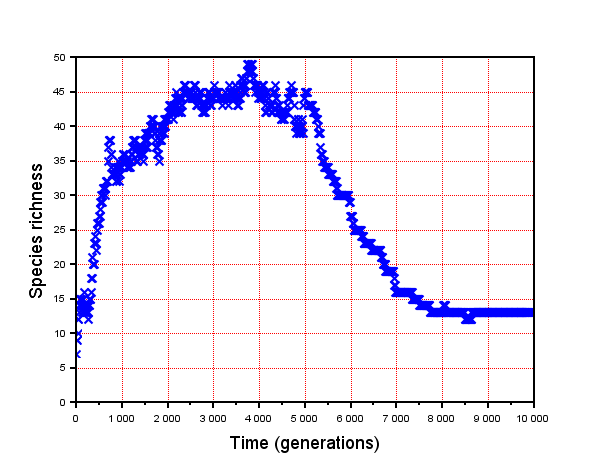 | 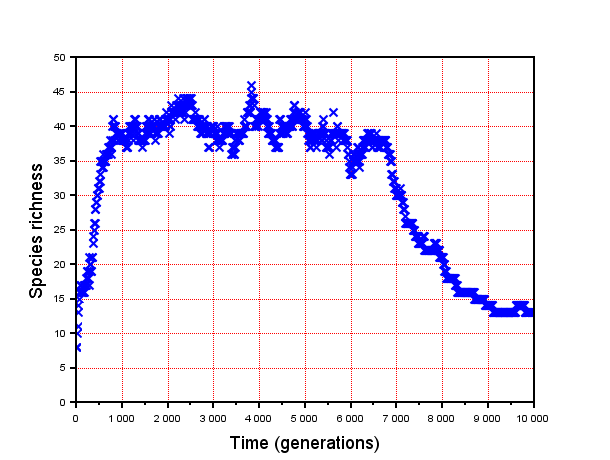 |

Table S3

Population dynamics in the total volume for the models varying by the time and the location of initial phage infestation of cells. Chemotaxis is on (see Additional files S2-S10).

|  | Early-time  (1^st^ generation) | Middle-time  (5000^th^ generation) | Late-time  (6600^th^ generation) |
| --- | --- | --- | --- |
| Into node (1,1) |  |  |  |
| Into node (3,3) |  |  |  |
| Into node (5,5) |  |  |  |

Table S4

Species richness (number of species) in the total volume for the models varying by the time and the location of initial phage infestation of cells. Chemotaxis is on (see Additional files S2-S10. Plots are made using the script from Additional files S11-S12).

|  | Early-time  (1^st^ generation) | Middle-time  (5000^th^ generation) | Late-time  (6600^th^ generation) |
| --- | --- | --- | --- |
| Into node (1,1) | 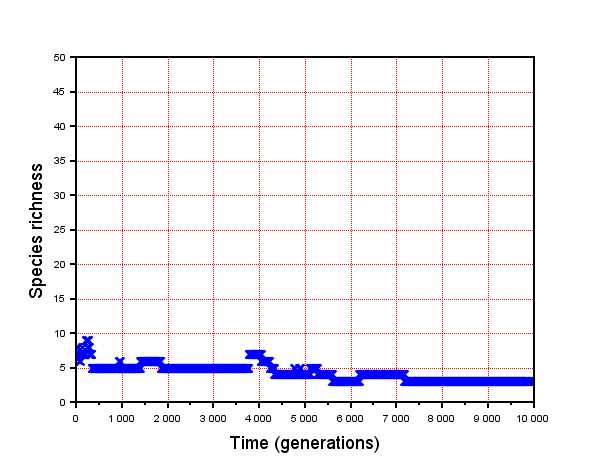 | 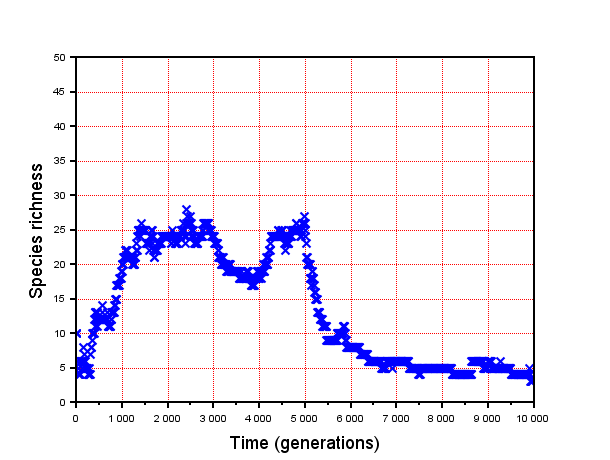 | 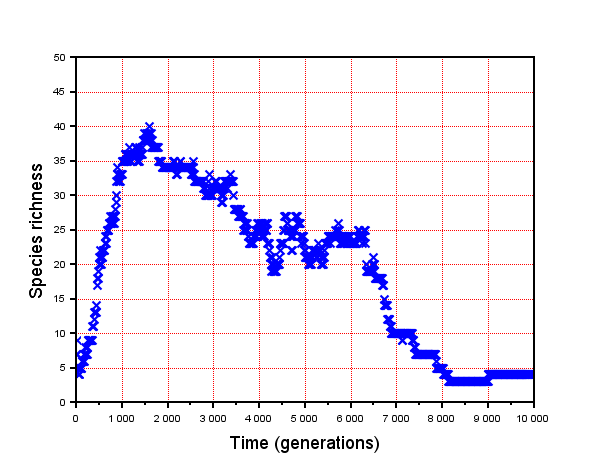 |
| Into node (3,3) | 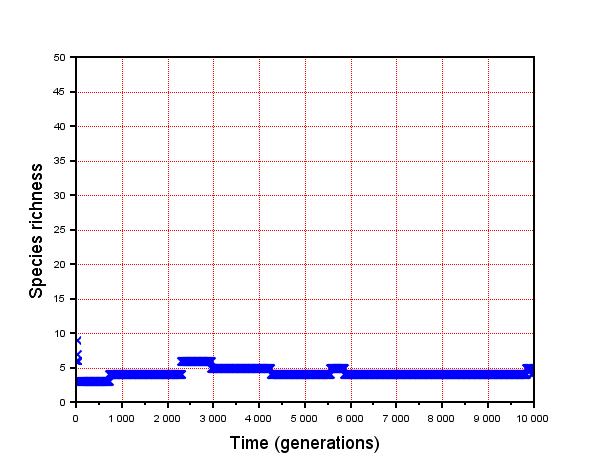 | 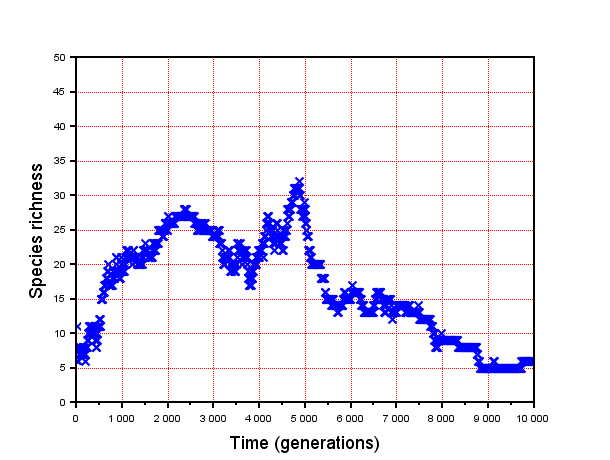 | 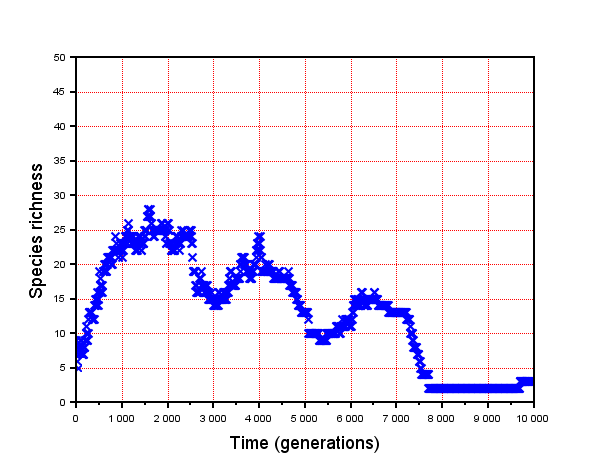 |
| Into node (5,5) | 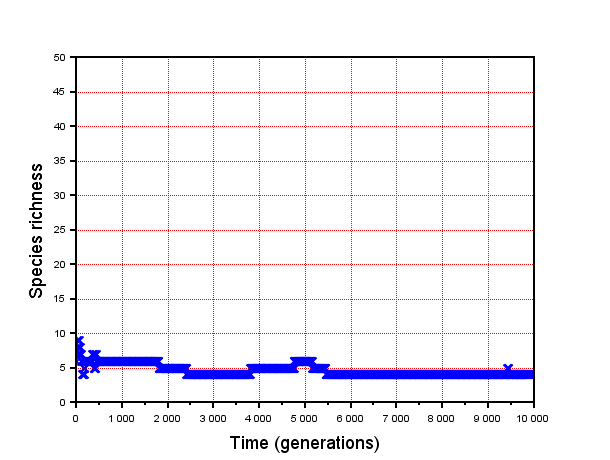 | 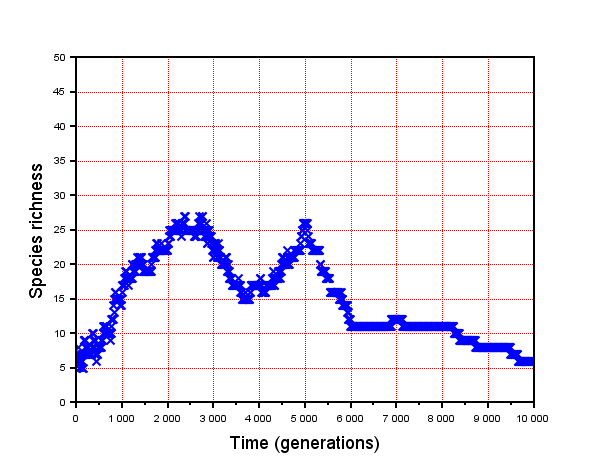 | 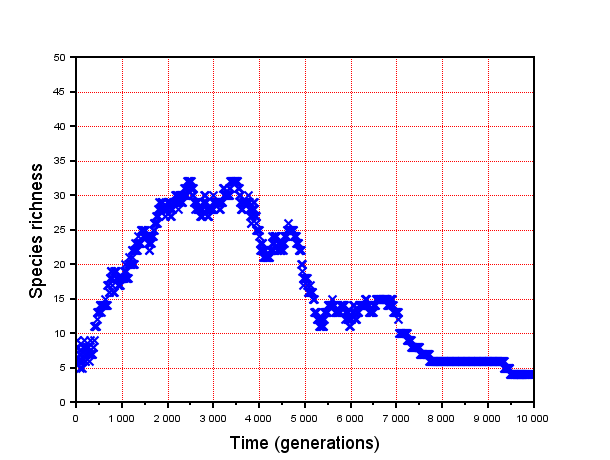 |
