# Supplementary material for: Erratum to: Bacteriophages affect evolution of bacterial communities in spatially distributed habitats: a simulation study
Source: BMC Microbiol. 2016 Apr 14;16:67. doi: 10.1186/s12866-016-0677-8 (PMC4831195; doi:10.1186/s12866-016-0677-8)
Supplement: Additional file 8: S1 — Archive containing the HEC executable file. 7-Zip archive containing the HEC executable file (Windows version). To switch chemotaxiss on pass 0.1 as a second command line parameter (the first is a model script). S2. HEC script 2D_phage.plentiful_edge.early.txt of early-time phage into (1,1) model. Text file with the model script of early-time phage added into (1,1) model. S3. HEC script 2D_phage.center.early.txt of early-time phage into (3,3) model. Text file with the model script of early-time phage added into (3,3) model. S4. HEC script 2D_phage.poor_edge.early.txt of early-time phage into (5,5) model. Text file with the model script of early-time phage added into (5,5) model. S5. HEC script 2D_phage.plentiful_edge.middle.txt of middle-time phage into (1,1) model. Text file with the model script of middle-time phage added into (1,1) model. S6. HEC script 2D_phage.center.middle.txt of middle-time phage into (3,3) model. Text file with the model script of middle-time phage added into (3,3) model. S7. HEC script 2D_phage.poor_edge.middle.txt of middle-time phage into (5,5) model. Text file with the model script of middle-time phage added into (5,5) model. S8. HEC script 2D_phage.plentiful_edge.late.txt of late-time phage into (1,1) model. Text file with the model script of late-time phage added into (1,1) model. S9. HEC script 2D_phage.center.late.txt of late-time phage into (3,3) model. Text file with the model script of late-time phage added into (3,3) model. S10. HEC script 2D_phage.poor_edge.late.txt of late-time phage into (5,5) model. Text file with the model script of late-time phage added into (5,5) model. S11. Data processing script speciesRichnessFluctuation.sce. A Scilab script that plots species richness dynamics. S12. A library SRlib.sce. A Scilab library sources used by such scripts as speciesRichnessFluctuation.sce, averageSRandBiomass.sce and speciation_rate.sce. (ZIP 594 kb) [file 12866_2016_677_MOESM8_ESM.zip › additional file 8 Extra supplementary/Extra supplementary material.docx]

**Additional files**

**Additional file S1**

**Title: Archive containing the HEC executable file**

**Description:** 7-Zip archive containing the HEC executable file (Windows version). To switch chemotaxiss on pass 0.1 as a second command line parameter (the first is a model script).

**Additional file S2**

**Title: HEC script 2D_phage.plentiful_edge.early.txt of early-time phage into (1,1) model**

**Description:** Text file with the model script of early-time phage added into (1,1) model.

**Additional file S3**

**Title: HEC script 2D_phage.center.early.txt of early-time phage into (3,3) model**

**Description:** Text file with the model script of early-time phage added into (3,3) model.

**Additional file S4**

**Title: HEC script 2D_phage.poor_edge.early.txt of early-time phage into (5,5) model**

**Description:** Text file with the model script of early-time phage added into (5,5) model.

**Additional file S5**

**Title: HEC script 2D_phage.plentiful_edge.middle.txt of middle-time phage into (1,1) model**

**Description:** Text file with the model script of middle-time phage added into (1,1) model.

**Additional file S6**

**Title: HEC script 2D_phage.center.middle.txt of middle-time phage into (3,3) model**

**Description:** Text file with the model script of middle-time phage added into (3,3) model.

**Additional file S7**

**Title: HEC script 2D_phage.poor_edge.middle.txt of middle-time phage into (5,5) model**

**Description:** Text file with the model script of middle-time phage added into (5,5) model.

**Additional file S8**

**Title: HEC script 2D_phage.plentiful_edge.late.txt of late-time phage into (1,1) model**

**Description:** Text file with the model script of late-time phage added into (1,1) model.

**Additional file S9**

**Title: HEC script 2D_phage.center.late.txt of late-time phage into (3,3) model**

**Description:** Text file with the model script of late-time phage added into (3,3) model.

**Additional file S10**

**Title: HEC script 2D_phage.poor_edge.late.txt of late-time phage into (5,5) model**

**Description:** Text file with the model script of late-time phage added into (5,5) model.

**Additional file S11**

**Title: Data processing script speciesRichnessFluctuation.sce**

**Description:** A Scilab script that plots species richness dynamics.

**Additional file S12**

**Title: A library SRlib.sce**

**Description:** A Scilab library sources used by such scripts as speciesRichnessFluctuation.sce, averageSRandBiomass.sce and speciation_rate.sce.
